# Probiotic potential of *Enterococcus faecalis* strains isolated from meconium

**DOI:** 10.3389/fmicb.2015.00227

**Published:** 2015-04-02

**Authors:** Ahmed K. Al Atya, Karima Drider-Hadiouche, Rozenn Ravallec, Amadine Silvain, Anne Vachee, Djamel Drider

**Affiliations:** ^1^Institut Charles Viollette, Polytech-Lille, Université Lille 1 – Sciences et Technologies, Villeneuve d’AscqFrance; ^2^Hôpital Victor Provo, RoubaixFrance; ^3^Génie Biologique et Alimentaire, Institut Charles Violette, ProbioGem Laboratoire, Polytech-Lille, Université Lille 1 – Sciences et Technologies, Villeneuve d’AscqFrance

**Keywords:** meconium, *Enterococcus faecalis*, antagonism, anti-*Staphylococcus* activity, enterocins

## Abstract

107 bacterial isolates with Gram positive staining and negative catalase activity, presumably assumed as lactic acid bacteria, were isolated from samples of meconium of 6 donors at Roubaix hospital, in the north of France. All these bacterial isolates were identified by MALDI-TOF mass spectrometry as *Enterococcus faecalis*. However, only six isolates among which *E. faecalis* 14, *E. faecalis* 28, *E. faecalis* 90, *E. faecalis* 97, and *E. faecalis* 101 (obtained from donor 3), and *E. faecalis* 93 (obtained from donor 5) were active against some Gram-negative bacteria and Gram-positive bacteria , through production of lactic acid, and bacteriocin like inhibitory substances. The identification of these isolates was confirmed by 16rDNA sequencing and their genetic relatedness was established by REP-PCR and pulsed field gel electrophoresis methods. Importantly, the aforementioned antagonistic isolates were sensitive to various classes of antibiotics tested, exhibited high scores of coaggregation and hydrophobicity, and were not hemolytic. Taken together, these properties render these strains as potential candidates for probiotic applications.

## Introduction

Human microbiome is undergoing dynamic changes in bacterial content in the gut during pregnancy and development of childhood (Dominguez-Bello et al., 2010). In early childhood, the presence of pathogenic species, or absence of beneficial ones, leads to adverse effects such as initiation of preterm birth (DiGiulio et al., 2008), and development of asthma, allergy, and autism (Hong et al., 2010; Johansson et al., 2011; Wang et al., 2011). Microbes colonizing the gastrointestinal tract of the infant are originated from the mother and surrounding environment during the birth and shortly thereafter (Mshvildadze et al., 2010). The fetus, as well as the intrauterine environment, have been considered sterile, but the presence of microbes was reported in amniotic fluid (Hitti et al., 1987), fetal membranes (Steel et al., 2005), and meconium (Gosalbes et al., 2013). Meconium is the first intestinal discharge of newborns, which consists of a viscous, sticky, dark green substance. It might contain materials ingested during time that the infant has spent in the uterus; these could include epithelial cells, mucus, bile acid, blood, lanugo, and amniotic fluid (Cleary and Wiswell, 1998; Gosalbes et al., 2013). Newborns seem to acquire their first microbiota at birth and maternal vaginal or skin bacteria colonize newborns delivered vaginally or by caesarian section (C-section; Alicea-Serrano et al., 2013). Hu et al. (2013) confirmed that meconium is not sterile and does contain diversified microbiota. Moles et al. (2013) characterized the microbiota from meconium and fecal samples obtained during the first 3 weeks of life from 14 donors. They showed that bacilli and other Firmicutes were important in meconium whilst Proteobacteria dominated in the fecal samples based on molecular method. *Staphylococcus* predominated in meconium while *Enterococcus* and Gram-negative bacteria (GNB) were more abundant in fecal samples (Moles et al., 2013). Makino et al. (2013) showed that several *Bifidobacterium* strains transmitted from the mother are colonizing the infant’s intestine shortly after birth. Thus, the gut of infant might contain different species such as Enterococci, Bifidobacteria, and Lactobacilli that protect mucus of infants from pathogenic species through production of inhibitory substances including hydrogen peroxide, organic acids, and bacteriocins (Vizoso-Pinto et al., 2006; Rodríguez et al., 2012). Enterococci, particularly *Enterococcus faecalis* and *E*. *faecium* are involved in the reduction or prevention of gastro intestinal tract infections (Franz et al., 2011). Enterococci belong to the group of LAB, which is known to produce lactic acid as the end product of sugar fermentation, and antimicrobials, which are active against pathogens including *Staphylococcus aureus* and *Listeria monocytogenes* (Campos et al., 2006). Further, many strains of *E. faecalis* produce bacteriocins named enterocins, a family of safe (Belguesmia et al., 2011), and ribosomally synthesized antimicrobial peptides (AMP; Drider and Rebuffat, 2011). This study aimed at studying and taking advantage of the LAB isolated from meconium sampled at Roubaix hospital in the North of France.

## Materials and Methods

### Preparation of Samples, Isolation, and Identification of Lactic acid Bacteria

Samples of fresh meconium were collected, in sterile dry plastic containers, from 6 donors (newborn infants), at Roubaix hospital in the North of France. Samples of meconium were stored at 4^∘^C until to be processed. One gram of meconium was resuspended in 9 ml of 0.9% (w/v) of sterile saline solution, and was serially diluted from 10^-1^ to 10^-6^. One ml of each dilution was poured onto Petri dishes and melt MRS (de Man-Rogosa-Sharpe) agar medium (Biokar, France; de Man et al., 1960) was poured gently. After agar solidification at room temperature, plates were incubated under 5% CO_2_ at 37^∘^C for 24–48 h. The grown colonies were Gram-stained and checked for catalase activity. Presumptive LAB strains were selected, maintained at -80^∘^C in MRS with 25% glycerol as stock culture, until use. The presumptive LAB isolates were identified by the VITEK MS v2.0 MALDI-TOF mass spectrometry according to manufacturer’s instructions.

### Antimicrobial Activity

The antibacterial activity was assessed against the GNB and Gram-positive bacteria (GPB) listed in **Table 1**, using the well known agar diffusion test (Naghmouchi et al., 2006). The cell free supernatants (CFS) used for antibacterial activity measurement were obtained by centrifuging (9000 ×*g*, 10 min, 4^∘^C), overnight cultures of *E. faecalis* grown at 37^∘^C for 18–24 h, on MRS broth. Wells were performed in solid agar and 50 μl of each CFS or neutralized cell-free supernatant (NCFS; pH6.5) were poured into the wells. The Petri plates were left at room temperature, in sterile conditions, for 1 h before incubation for 18 h at adequate temperature. After this period of incubation, the antibacterial activity was detected by observing the inhibition zones around the well containing the CFS or NCFS. Importantly, the impacts of temperature and pH variations as well as proteinase K, papain, trypsin, and α-chymotrypsin at 1 mg/ml (Sigma-Aldrich Germany) were determined toward *Listeria innocua* CIP 103982.

**Table 1:** Inhibitory activity before neutralization of the cell-free supernatant (BN) and after the neutralization of the cell-free supernatant (AN).

Indicator strain	*Enterococcus faecalis 14*		*E. faecalis* 28		*E. faecalis* 90		*E. faecalis* 93		*E. faecalis* 97		*E. faecalis* 101	
	BN	AN	BN	AN	BN	AN	BN	AN	BN	AN	BN	AN
*Listeria monocytogenes* ATCC 3512	7.15 ± 0.2	5.8 ± 0.4	6.0 ± 0.2	3.6 ± 0.84	5.9 ± 0.5	3.55 ± 08	6.6 ± 0.1	4.0 ± 0.1	6.25 ± 0.4	3.70 ± 0.6	6.5 ± 0.5	3.3 ± 0.24
*Listeria innocua* CIP103982	7.65 ± 0.2	6.3 ± 0.4	6.1 ± 0.1	4.8 ± 0.56	6.2 ± 0.2	4.3 ± 0.6	5.7 ± 0.1	4.1 ± 0.5	6.3 ± 0.9	4.20 ± 0.2	7.5 ± 0.7	5.6 ± 0.8
*Bacillus subtilis* ATCC 6633	7.7 ± 0.4	6.2 ± 0.1	6.8 ± 0.2	4.7 ± 0.3	7.3 ± 0.2	4.8 ± 0.3	6.2 ± 46	3.8 ± 0.1	5.5 ± 0.7	2.60 ± 0.5	7.2 ± 0.2	3.3 ± 0.24
*Staphylococcus aureus* ATCC33862	4.8 ± 0.3	3.6 ± 0.8	6.0 ± 0.2	5.6 ± 0.47	6.1 ± 0.1	5.5 ± 0.6	5.7 ± 0.9	4.9 ± 0.9	4.6 ± 0.21	4.50 ± 0.5	4.8 ± 0.2	3.6 ± 0.3
Methicillin-resistant* S. aureus* (MRSA)	2.6 ± 0.7	0	3.2 ± 0.8	0	2.8 ± 0.5	0	2.5 ± 0.4	0	2.3 ± 0.55	0	2.4 ± 0.6	0
*Enterococcus faecalis* ATCC	7.6 ± 0.56	6.1 ± 0.8	5.2 ± 0.4	4.20 ± 0.6	5.1 ± 0.3	4.2 ± 0.6	5.8 ± 3	4.3 ± 1.0	4.7 ± 0.14	5.10 ± 0.9	4.5 ± 0.4	5.0 ± 0.80
*Escherichia coli* CIPI103982	6.4 ± 0.2	0	4.85 ± 0.2	0	4.5 ± 0.5	0	4.3 ± 0.7	0	3.9 ± 0.25	0	4.15 ± 0.	0
Klebsiella oxytoca*	6.65 ± 0.3	0	5.65 ± 0.3	0	4.7 ± 0.1	0	4.6 ± 0.4	2.1 ± 0.3	3.75 ± 0.1	0	4.7 ± 0.1	2.55 ± 0.20
*Proteus mirabili*s*	5.4 ± 0.3	0	5.95 ± 0.5	0	6.25 ± 0	0	3.9 ± 0.1	0	4.45 ± 0.4	0	5.0 ± 15	0
*Salmonella* Heidelberg*	5.7 ± 0.3	0	5.15 ± 0.1	0	4.7 ± 0.7	0	5.3 ± 0.7	0	4.55 ± 0.0	0	4.15 ± 0.0	0
Pseudomonas fluorescens*	5.8 ± 0.4	0	5.0 ± 0.25	0	5.6 ± 0.5	2.5 ± 0.3	5.5 ± 0.5	0	4.4 ± 0.15	0	6.2 ± 0.1	2.6 ± 0.20
*Candida albican*s ATCC10231	0	0	0	0	0	0	0	0	0	0	0	0
*Saccharomyces cerevisiae*^∗∗^	0	0	0	0	0	0	0	0	0	0	0	0

The diameter of inhibition zone is measured in millimeters. The value “0” designs the absence of inhibitory activity under the experimental conditions used in this work. The SD designs the standard deviation. The data are the means of at least three independent assay. Each assay consisted in three measures. * Designs strains obtained from our laboratory collection, while ^∗∗^ designs strain generously provided by Lesaffre company.

### Molecular Characterization of the Antagonistic Isolates

Total DNA was extracted from each antagonistic isolates using Wizard®; Genomic DNA Purification Kit (Promega, Madison, WI, USA). For 16S rDNA analysis, total DNA was amplified with 16S forward 5′-AGAGTTTGATCMTGGCTCAG-3′ and 16S reverse 5′-GGMTACCTTGTTACGAYTTC-3′ primers (Drago et al., 2011), and the following PCR program: 94^∘^C/3 min, 29 cycles at 94^∘^C/40 s, 55^∘^C/50 s and 72^∘^C/2 min; and finally 72^∘^C/10 min. For REP-PCR analysis, total DNA was amplified with 5′- (GTG)_5_-3 primer and the following program: 95^∘^C/5 min, 30 cycles at 94^∘^C/1 min, 40^∘^C/1 min, 72^∘^C/8 min, and finally 72^∘^C/16 min. Amplicons were separated on 1% agarose gel. Electrophoresis was performed at 100 V for 2 h using 1X Tris-borate-EDTA. The gels were stained with GEL-RED (Biotium, Canada), and visualized by GelDoc (Bio-Rad, France). Dendrograms were generated automatically using the DIVERSILAB®; software (Biomérieux, Craponne, France).

The amplicons for 16S rDNAs were purified with the pJET1Kit (Fermentas, France), and sequenced at Eurofins MWG Operon (Germany). Partial 16S rDNAs sequences were compared to that of *E. faecalis* ATCC 19433 using ClustalW2 software^1^.

### Challenge Tests: *Staphylococcus aureus* ATCC 33862 vs. Antagonistic *E. faecalis*

*Enterococcus faecalis* was grown in MRS and *S. aureus* ATCC33862 in Brain Heart Infusion (BHI; 10^5^ CFU/ml) at 37^∘^C for 18 h. After this period, the cultures had reached 10^8^ CFU/ml for *E. faecalis*, and 10^5^ CFU/ml for *S. aureus* ATCC33862. Afterward, 0.1 ml of each culture was added to 20 ml of BHI containing leading to 5. 10^5^ CFU/ml (*E. faecalis*) and 5. 10^2^ CFU/ml (*S. aureus* ATCC33862). One ml of this coculture was withdrawn at 0, 4, 8, 12, 18, and 24 h, and was serially diluted from 10^-1^ to 10^-6^, in 0.9% (w/v) of saline water. Then 1 ml of each dilution was plated on Plates dishes containing MRS agar for *E. faecalis*, and Chapman agar for *S. aureus* ATCC33862. The number of colonies was determined after 24 h of incubation at 37^∘^C.

### Lactic Acid Quantification

Lactic acid was quantified by HPLC spectra system P1000XR (Thermo, USA). Isolates *E. faecalis* 28 and *E. faecalis* 93 were grown in MRS broth at 37^∘^C, samples were withdrawn after 0, 4, 8, 12, 18, and 24 h of incubation, centrifuged (10,000 ×x *g*, 10 min, 4^∘^C), and sterilized by filtration using Millipore filter (0.2 μm). The CFS were divided into 3 samples. Sample 1 was used to measure pH, sample 2 to assess the antibacterial activity against *S. aureus* ATCC33862 by the well diffusion method (Naghmouchi et al., 2006), and sample 3 to determine the concentration of lactic acid. All these experiments were performed in triplicate.

### Autoaggregation Assay

Autoaggregation assays were performed according to initial protocol of Del Re et al. (2000) and modified by Al Kassaa et al. (2014). Thus, isolates of interest were cultured on MRS for 18 h at 37^∘^C, then cells were centrifuged (5,000 ×*g*, 4^∘^C, 15 min), washed three times with phosphate saline (PBS) buffer (pH7.0) and were resuspended in sterile PBS to obtain a viable cell count of 10^8^ CFU/ml. The bacterial suspension of 4 ml was mixed by vortexing for 10 s and incubated at room temperature for different time intervals (0, 1, 2, 3, 4, or 5 h). At each interval, the growth was measured at OD_600__nm_. The percentage of autoaggregation was expressed as follows:

$$\%\,autoaggregation\,=[(OD_{1}-OD_{2})/\left(OD_{1}\right)]\times 100.$$

Where OD_1_ represents the optical density at time (1, 2, 3, 4, or 5 h) and OD_2_ the data at time 0 h. All experiments were performed in triplicate.

### Hydrophobicity

The hydrophobicity was determined using *in vitro* method to detect the bacterial adhesion to hydrocarbons (Rosenberg et al., 1980). Each antagonistic LAB isolate was grown in MRS broth at 37^∘^C for 18 h. The bacterial cells harvested by centrifugation (5,000 ×*g*, 4^∘^C, 15 min) were washed twice with PBS (pH7.0), resuspended in the same solution and the OD_600__nm_ was determined. One milliliter of xylene (Fluka, Germany) was added to 3 ml of cell suspension and vortexed for 2 min after 10 min of incubation at room temperature. The aqueous phase was removed after 2 h of incubation at room temperature and the OD_600__nm_ was determined. The percentage of hydrophobicity was calculated using the formula given below. All experiments were performed in triplicate.

$$\%\,hydrophobicity\,=[(OD_{600\,nm}\,reading\,1-OD_{600\,nm}\,reading\,2)/OD_{600\,nm}\,reading\,1]\times 100.$$

### Hemolytic Activity

The hemolytic activity was determined by streaking Enterococcal isolates on Columbia blood agar supplemented with 5% (v/v) of human blood or Sheep blood. The plates were incubated at 37^∘^C for 48 h. The presence or absence of zones of clearing around the colonies was interpreted as β-hemolysis (positive hemolytic activity) or χ-hemolysis (negative hemolytic activity), respectively. When observed, greenish zones around the colonies were interpreted as α-hemolysis and taken as negative for the assessment of hemolytic activity (Semedo et al., 2003).

### Antimicrobial Susceptibility

Study of antibiotic susceptibility was performed by three independent methods: disk diffusion method, minimal inhibitory concentrations (MICs) using *E* test (Bio-Mérieux, France), and VITEK 2 system (Bio-Mérieux, France). Related to this, AST-P606 card was used for “Enterococci” encompassing nearly all important antibiotics among which ampicillin, gentamicin, kanamycin, streptomycin, levofloxacin, moxifloxacin, erythromycin, clindamycin, linezolid, teicoplanin, vancomycin, tetracycline, nitrofurantoin chloramphenicoland trimethoprim-sulfamethoxazole. Antibiotic susceptibility and MICs were determined and analyzed according to the French Committee on Antimicrobial Susceptibility Testing (CA-SFM 2013).

## Results

### Scavenging of Lactic Acid Bacteria and Elucidation of their Antagonism

Hundred and seven LAB isolates were isolated from 6 samples of meconium newborns infants (6 donors) obtained at Roubaix hospital in the North of France. The number of LAB isolates per sample of meconium/donor was as follows: sample 1 (20 LAB isolates), sample 2 (12 LAB isolates), sample 3 (LAB 26 isolates), sample 4 (LAB 12 isolates), sample 5 (LAB 17 isolates), and sample 6 (LAB 20 isolates). In addition to their Gram positive staining, and absence of catalase activity, all these 107 LAB isolates were able to hydrolyze esculine. Importantly, all of them were identified by MALDI TOF mass spectrometry (Biomérieux, France), with high score (>99%), as *E. faecalis.* Among these *E. faecalis* species, only six isolates designed as *E. faecalis* 14, *E. faecalis* 28, *E. faecalis* 90, *E. faecalis* 93, *E. faecalis* 97, and *E. faecalis* 101 resulted to be antagonistic. Partial 16S ribosomal DNA sequences of these isolates as well as those of *E. faecalis* ATCC 19433 were aligned using ClustalW2 software^2^. This alignment shows a percentage of similarity higher than 99.7% to *E. faecalis*, confirming phylogenetic identification of our isolates. When the 16S rDNA sequence of *E. coli* ATCC 11229 was compared to that of enterococci, the percentage of similarity was estimated to 76.33%, which clearly is very low. These antagonistic isolates were able to produce lactic acid and bacteriocin like inhibitory substances (BLIS) and consequently to inhibit growth of GNB and GPB (**Table 1**). This antagonism was restricted to GPB when the pH of CFS was adjusted to 6.5 (**Table 1**). The BLIS produced by the aforementioned antagonistic strains resulted to be insensitive to pH and temperature variations but sensitive to proteases. Remarkably, only *E. faecalis* 90 and *E. faecalis* 101 were active against *Pseudomonas fluorescens* (**Table 1**).

### Genetic Patterns of the Antagonistic Strains

**FIGURE 1:**
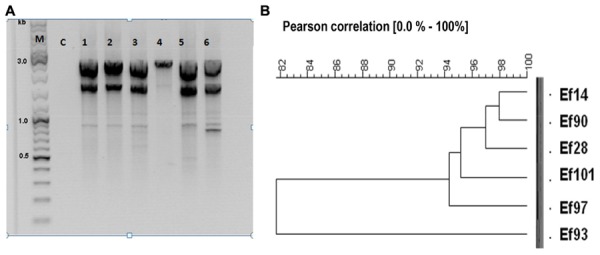
**Genetic relatedness of antagonistic strains. (A)** REP-PCR products were separated on 1% agarose gel. Lane M corresponds to the DNA markers (Thermo, USA), lane C corresponds to the PCR control (PCR carried without DNA template), lanes 1, 2, 3, 4, 5, 6 correspond to genetic patterns of *Enterococcus faecalis* 14 (Ef14), *E. faecalis* 28 (Ef28), *E. faecalis* 90 (Ef90), *E. faecalis* 93 (Ef93), *E. faecalis* 97 (Ef97), and *E. faecalis* 101 (Ef101). **(B)** Dendrogram outlining Pearson correlation of antagonistic *E. faecalis* 14 (Ef14), *E. faecalis* 28 (Ef28), *E. faecalis* 90 (Ef90), *E. faecalis* 93 (Ef93), *E. faecalis* 97 (Ef97) and *E. faecalis* 101 (Ef101).

Genetic patters gathered by REP-PCR permitted to create two main groups. Indeed, group 1 contains isolates *E. faecalis* 14, *E. faecalis* 28, *E. faecalis* 90, *E. faecalis* 97, and *E. faecalis* 101, whereas group 2 contains only isolate *E. faecalis* 93 (**Figure 1**). Based on their genetic patterns and analysis of the dendrogram and pulsed field gel electrophoresis (PFGE; data not shown here), we assume that *E. faecalis* 14 and *E. faecalis* 28, then *E. faecalis* 90 and *E*. *faecalis* 93, *E. faecalis* 97 and *E. faecalis* 101 have similar DNA patterns.

### Highlights on Anti-*S. aureus* ATCC33862 Activity

**FIGURE 2:**
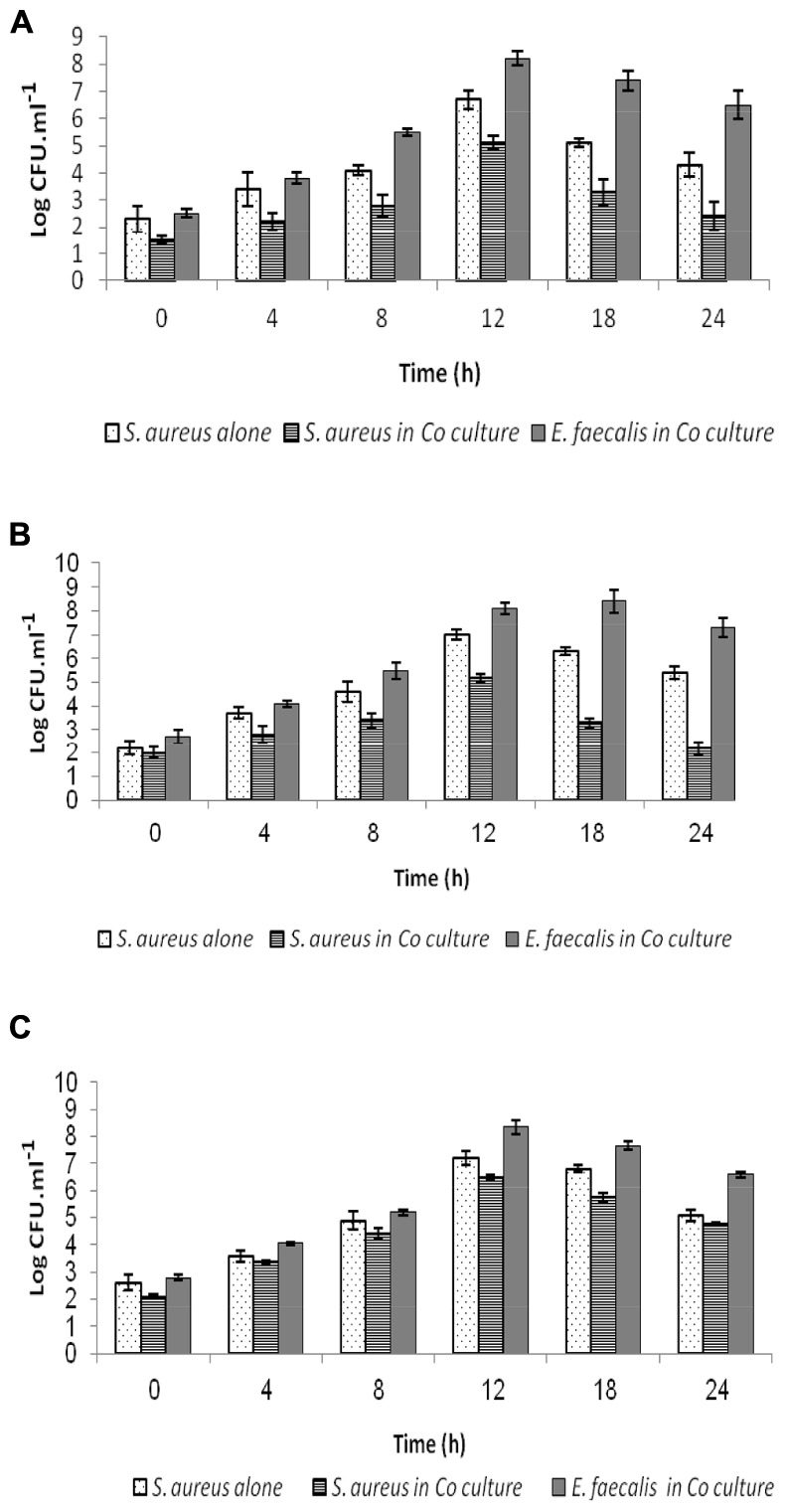
**Interplay between *Staphylococcus aureus* ATC C33862 and enterococci during coculture.** Samples were withdrawn at regular intervals of time and plated onto agar Brain Heart for counting of *S. aureus* and on agar MRS for counting of enterococci *E. faecalis* 28 **(A)**, *E. faecalis* 93 **(B)**, and *E. faecalis* ATCC 29212 **(C)**. The values expressed in Log CFUT ml^-1^ (±SD) are the average of at least three independent experiments.

The data gathered from the challenge tests indicate that inhibition of *S. aureus* ATCC 33862 by *E. faecalis* 28 and *E. faecalis* 93 was due to production of lactic acid and bacteriocin. The number of *S. aureus* ATCC33862 cells has decreased of about 5.5 Log, 5 Log, and 2 log in presence of *E. faecalis* 28, *E. faecalis* 93, and *E. faecalis* ATCC29212 (**Figures 2A–C**).

### Production of Lactic Acid and Bacteriocins like Inhibitory Substances

**FIGURE 3:**
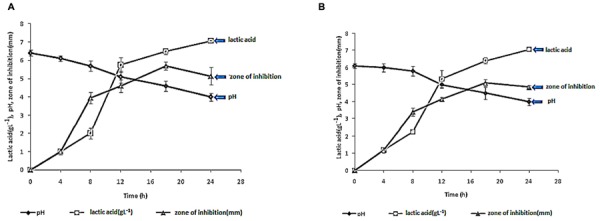
**Measurement of pHs, amount of lactic acid (g L^-1^) and inhibitory activity of the cell free supernatants (CFS) prepared from *E. faecalis* 28 **(A)** and *E. faecalis* 93 **(B)** after different times of growth.** The data are the average of at least three independent experiments.

*Enterococcus faecalis* 28 and *E. faecalis* 93 isolates produced up to 7.06 g/l of lactic acid, after 24 h of culture. As shown on **Figures 3A,B**, the drop of pH observed during coculture, was correlated to increase of lactic acid production. The anti-staphylococcal activity was ascribed to both lactic acid and bacteriocins, and this activity appeared to increase until 18 h of co-culture.

### Cell Surface Properties of the Antagonistic Isolates

**Table 2** shows the scores of hydrophobicity and autoaggregation of antagonistic isolates. The highest scores were registered for *E. faecalis* 14; that was isolated from donor 3.

**Table 2:** Autoaggregation and hydrophobicity percentages of antagonistic meconium enterococci.

Isolates	Autoaggregation (%)	Hydrophobicity (%)
*E. faecalis* 14	49 ± 0.47	47 ± 0.8
*E. faecalis* 28	44 ± 1.6	45.6 ± 0.47
*E. faecalis* 90	35 ± 1.24	41 ± 1.2
*E. faecalis* 93	44.6 ± 1.8	46.3 ± 1.2
*E. faecalis* 97	41 ± 1.24	34 ± 1.4
*E. faecalis* 101	45 ± 1.6	43 ± 0.94
*E. faecalis* ATCC29212	45.6 ± 0.1.2	42.3 ± 0.9

### Antagonistic Strains are Sensitive to Antibiotics and are Not Hemolytic

As indicated in **Tables 3A,B**, antagonistic strains are sensitive to antibiotics tested. To be noted that E. faecalis 14 and E. faecalis 93 are resistant to erythromycin, whilst isolates E. faecalis 28, E. faecalis 90, E. faecalis 97, and E. faecalis 101 exhibit intermediate resistance to this drug. Further, all antagonistic isolates are sensitive to ampicillin, gentamicin, kanamycin, streptomycin, levofloxacin, moxifloxacin, linezolid, teicoplanin, vancomycin, tetracycline, nitrofurantoin, and chloramphenicol but are resistant to clindamycin and trimethoprim-sulfamethoxazole. The data analysis was performed according to “Antibiogram committee of French Microbiology of Society” based on the European Committee on Antimicrobial Susceptibility Testing recommendations. Remarkably, these strains were not hemolytic (data not shown).

**Table 3A:** Antibiotic susceptibility of antagonistic isolates.

Antibiotics MIC: mg/l	*E. faecalis* 14	*E. faecalis* 28	*E. faecalis* 90	*E. faecalis* 93	*E. faecalis* 97	*E. faecalis* 101
Ampicillin	S ( ≤ 2)	S ( ≤ 2)	S ( ≤ 2)	S ( ≤ 2)	S ( ≤ 2)	S ( ≤ 2)
Gentamicin (high level)	S	S	S	S	S	S
Kanamycin (high level)	S	S	S	S	S	S
Streptomycin (high level)	S	S	S	S	S	S
Levofloxacin	S (1)	S (1)	S (1)	S (1)	S (1)	S (1)
Moxifloxacin	S ( ≤ 0.25)	S ( ≤ 0.25)	S ( ≤ 0.25)	S ( ≤ 0.25)	S ( ≤ 0.25)	S ( ≤ 0.25)
Clindamycin	R ( ≥ 8)	R ( ≥ 8)	R ( ≥ 8)	R ( ≥ 8)	R ( ≥ 8)	R ( ≥ 8)
Linezolid	S (2)	S (2)	S (2)	S (2)	S (2)	S (2)
Teicoplanin	S ( ≤ 0.5)	S ( ≤ 0.5)	S ( ≤ 0.5)	S ( ≤ 0.5)	S ( ≤ 0.5)	S ( ≤ 0.5)
Vancomycin	S (1)	S (1)	S (1)	S (2)	S (1)	S (2)
Tetracyclin	S ( ≤ 1)	S ( ≤ 1)	S ( ≤ 1)	S ( ≤ 1)	S ( ≤ 1)	S ( ≤ 1)
Nitrofurantoin	S ( ≤ 16)	S ( ≤ 16)	S ( ≤ 16)	S ( ≤ 16)	S ( ≤ 16)	S ( ≤ 16)
Chloramphenicol	S (8)	S (8)	S (8)	S (8)	S (8)	S (8)
Trimethoprime/sulfamethoxzole	S ( ≤ 10)	S ( ≤ 10)	S ( ≤ 10)	S ( ≤ 10)	S ( ≤ 10)	S ( ≤ 10)
Erythromycin	R ( ≥ 8)	I (4)	I (4)	R ( ≥ 8)	I (4)	I (4)

R, Resistant; S, Sensitive; I, Intermediate; MIC, Minimal inhibitory concentration determined by Vitek 2 system.

**Table 3B:** Antibiotic susceptibility of antagonistic isolates.

Antibiotics MIC: mg/l	*E. faecalis* 14	*E. faecalis* 28	*E. faecalis* 90	*E. faecalis* 93	*E. faecalis* 97	*E. faecalis* 101
Ampicillin	S (0.75)	S (0.75)	S (0.75)	S (0.75)	S (0.38)	S (0.38)
Gentamicin (high level)	S	S	S	S	S	S
Kanamycin (high level)	S	S	S	S	S	S
Streptomycin (high level)	S	S	S	S	S	S
Levofloxacin	S (1)	S (1)	S (1)	S (1)	S (1)	S (1)
Moxifloxacin	S	S	S	S	S	S
Clindamycin	R	R	R	R	R	R
Linezolid	S (2)	S (1)	S (1)	S (1.5)	S (1.5)	S (2)
Teicoplanin	S (0.25)	S (0.25)	S (0.25)	S (0.25)	S (0.25)	S (0.25)
Vancomycin	S (2)	S (1)	S (1)	S (1)	S (2)	S (2)
Tetracyclin	S	S	S	S	S	S
Nitrofurantoin	S	S	S	S	S	S
Chloramphenicol	S	S	S	S	S	S
Trimethoprime/sulfamethoxzole	S	S	S	S	S	S
Erythromycin	R (2)	I (2)	I (1)	R (1.5)	I (1)	I (1.5)

R, Resistant; S, Sensitive; I, Intermediate determined by Vitek 2; MIC, Minimal inhibitory concentration determined by *E* test.

### Accession Numbers

The accession numbers were KP057871 (*E. faecalis* 14), KP057872 (*E. faecalis* 28*),* KP057873* (E. faecalis* 90*),* KP057874 (*E. faecalis* 93*)*, KP057875 (*E. faecalis* 97), KP057876 (*E. faecalis* 101), NR_115765.1 (*E. faecalis* ATCC 19433), and GQ340751.1 *Escherichia coli* ATCC 11229.

## Discussion

This study aimed at strictly isolating LAB and particularly bacteriocinogenic LAB from meconium of newborns infants. Thus, 107 bacterial isolates were obtained from meconium of 6 donors at Roubaix Hospital in the north of France. All the bacterial isolates were identified as *E. faecalis*. Interestingly, Borderon et al. (1996), Adlerberth et al. (2006) showed that *E. faecalis* colonizes the gut of vaginally and cesarean-delivered term and preterm neonates Clinical isolates of enterococci are weakly diverse comparatively to those obtained from the environment, but *E. faecalis* remains the dominant species (Kuhn et al., 2003). According to Fisher and Phillips (2009), this weak diversity could be linked to the virulence factors present in this species.

Isolates *E. faecalis* 14, *E. faecalis* 28, *E. faecalis* 90, *E. faecalis* 93, *E. faecalis* 97, and *E. faecalis* 101 produce inhibitory compounds that comprise lactic acid and BLIS. The concomitant production of these antimicrobials inhibited a set of GNB and GPB, including the robust methicillin-resistant *S. aureus* (MRSA), which is frequently encountered in hospital environment. Remarkably, this antagonism was abolished when the CFS was neutralized, highlighting thereof the role of BLIS. Vancomycin is the standard antibiotic used for the treatment of MRSA infections. Recently, Liang et al. (2014), suggested daptomycin as a reasonable alternative for treating MRSA mainly for osteoarticular infections (OAIs). The activity of these antibiotics could be potentialized with bacteriocins, which are overall safe (Belguesmia et al., 2011), ribosomally synthesized AMP (Drider and Rebuffat, 2011). Genotyping of these isolates performed by REP-PCR and PFGE (data not shown) concluded to three different strains. Percent Identity Matrix based on 16S ribosomal DNA confirms a high score of similarity of enterococci sequences and a low score of similarity with *E. coli* ATCC 11229 used as different bacterial taxon.

In this study, *E. faecalis* 28 and *E. faecalis* 93, obtained from two different sources, were the most actives isolates. In their presence, the counts of *S. aureus* ATCC33862 has drastically decreased. Further, the antagonistic isolates displayed high scores of autoaggregation and hydrophobicity. These two characteristics might be important for the interplay of these isolates with *S. aureus* ATCC33862. Clewell et al. (2002) reported that production of aggregation substance (Agg) on the donor cell-surface facilitates contact with recipient cell by binding to enterococcal binding substance.

Importantly, these antagonistic strains were not hemolytic and resulted to be sensitive to antibiotic usually used to treat enterococci infections. The resistance to clindamycin and Trimethoprime/sulfamethoxzole is thought to be a species characteristic. According to the opinion of the FEEDAP panel on the updating of criteria used in the assessment of bacteria for resistance to antibiotics of human and veterinary importance, strains carrying an acquired resistance to antimicrobial(s) should not be used as feed additives, unless it can be demonstrated that it is a result of chromosomal mutation(s) (European Food Safety Authority [EFSA], 2005).

This study unveiled the presence of only *E. faecalis* species in samples of meconium obtained from six donors at Roubaix hospital in the north of France. Off 107 *E. faecalis* isolates, only *E. faecalis* 14, *E. faecalis* 28, *E. faecalis* 90, *E. faecalis* 93, *E. faecalis* 97, and *E. faecalis* 101 resulted to be bacteriocinogenic. In the best of our knowledge, this is the first report dealing with bacteriocinogenic LAB from meconium. The number of antagonistic isolates is relatively low (5.60%) compared to the frequency usually reported in the literature (Nes et al., 2014). The high hydrophobicity and aggregation scores registered for the aforementioned antagonistic isolates, as well as absence of hemolytic activity and sensibility to various antibiotic render these strains as potential candidates for probiotic applications.

## Conflict of Interest Statement

The authors declare that the research was conducted in the absence of any commercial or financial relationships that could be construed as a potential conflict of interest.

## Footnotes

1 http://www.ebi.ac.uk/Tools/msa/clustalw2/

2 http://www.ebi.ac.uk/Tools/msa/clustalw2/
